# A Versatile Molecular Tagging Method for Targeting Proteins to Avian Reovirus muNS Inclusions. Use in Protein Immobilization and Purification

**DOI:** 10.1371/journal.pone.0013961

**Published:** 2010-11-12

**Authors:** Alberto Brandariz-Nuñez, Rebeca Menaya-Vargas, Javier Benavente, Jose Martinez-Costas

**Affiliations:** Department of Biochemistry and Molecular Biology, Faculty of Pharmacy and Center for Research in Biological Chemistry and Molecular Materials, University of Santiago de Compostela, Santiago de Compostela, Spain; University of Crete, Greece

## Abstract

**Background:**

Avian reoviruses replicate in viral factories, which are dense cytoplasmic compartments estabilished by protein-protein interactions. The non-structural protein muNS forms the factory scaffold that attracts other viral components in a controlled fashion. To create such a three-dimensional network, muNS uses several different self-interacting domains.

**Methodology/Principal Findings:**

In this study we have devised a strategy to identify muNS regions containing self-interacting domains, based on the capacity of muNS-derived inclusions to recruit muNS fragments. The results revealed that the muNS region consisting of residues 477–542 was recruited with the best efficiency, and this raised the idea of using this fragment as a molecular tag for delivering foreign proteins to muNS inclusions. By combining such tagging system with our previously established method for purifying muNS inclusions from baculovirus-infected insect cells, we have developed a novel protein purification protocol.

**Conclusions/Significance:**

We show that our tagging and inclusion-targeting system can be a simple, versatile and efficient method for immobilizing and purifying active proteins expressed in baculovirus-infected cells. We also demonstrate that muNS inclusions can simultaneously recruit several tagged proteins, a finding which may be used to generate protein complexes and create multiepitope particulate material for immunization purposes.

## Introduction

Avian reoviruses are fusogenic viruses that belong to the *Orthoreovirus*, one of the twelve genera of the *Reoviridae* family [1], [2]. They are pathogenic viruses involved in several syndromes that affect poultry [3], [4]. Avian reovirus replicates in the cytoplasm and is one of the few non-enveloped viruses that are able to induce fusion of infected cells [5]. The viral genome is composed of 10 segments of double-stranded RNA, which are enclosed within a double-layered protein capsid with an external diameter of 85 nm and icosahedral symmetry. Details of avian reovirus structure, protein composition and replicative cycle have been described elsewhere [6], [7], [8].

Avian reoviruses replicate within cytoplasmic globular inclusions termed viral factories. These structures contain viral structural and non-structural proteins, together with viral RNA, but they lack cell organelles and membranes [9], [10]. The expression of individual proteins by cell transfection revealed that the non-structural protein muNS is the only viral protein that forms cytoplasmic inclusions in the absence of any other viral factor [10]. These muNS-derived inclusions are very similar to the native viral factories, suggesting that this protein forms the basic scaffold of the factories in avian-reovirus infected cells. Analysis of transfected cells co-expressing muNS and other viral proteins revealed that muNS plays an important role in the early steps of viral morphogenesis by temporally and selectively controlling the recruitment of specific viral proteins to viral factories [9].

We have recently carried out an extensive characterization of inclusion formation by avian reovirus muNS [11]. We found, in clear contrast with the situation reported for mammalian reoviruses and many other animal viruses [12], [13], [14], that neither ARV-derived factories nor muNS-derived inclusions are associated to the cytoskeleton, their formation and evolution are not dependent on the microtubule network, and are not related to aggresome or autophagosome generation. By two-hybrid analysis, we demonstrated that muNS monomers have the ability to self-associate. We also developed a simple method for purifying the inclusions made by muNS in baculovirus-infected cells, and the analysis of their protein composition indicated that muNS is the main building block of these cytoplasmic globular structures.

Analysis of the domain composition of the 635-residue muNS protein produced the following results: i) the region comprising residues 448 to 635 constitutes the minimal muNS portion able to form inclusions; we designated it muNS-Mi. ii) muNS-Mi is composed of four differentiated domains: two predicted coiled-coil elements that we termed Coil1 (C1; residues 448 to 477) and Coil2 (C2; residues 539 to 605), a stretch of amino acids linking both coiled-coils that we termed Intercoil (IC; residues 477 to 542), and a C-terminal part of the protein that we termed C-Tail (CT; residues 605 to 635).

We also investigated the contribution of the four muNS-Mi domains to inclusion-forming activity and determined that all of them are essential for inclusion formation. Domain C1 can be replaced by exogenous dimeric domains, and CT plays an important role in orienting the muNS inter-monomer contacts to form basal oligomers as well as influencing inclusion shape and inclusion formation efficiency. We also identified an additional domain located at the N-terminus of muNS, which is not essential for inclusion formation, but plays a role in inclusion maturation.

The original aim of this study was to develop an alternative method for detecting interactions between the different muNS domains. Towards this end, we analyzed the ability of individual muNS domains to get incorporated into cytoplasmic inclusions formed by muNS in CEF. The domains that were most efficiently incorporated into inclusions were the N-terminal part of the protein and IC. This information was then used to develop a method that used IC as a molecular tag. We demonstrate the validity of our system by purifying proteins that remained active while integrated inside muNS-derived protein inclusions. We show that our method can be used to purify soluble and inclusion-integrated active proteins. We also show that muNS-inclusions have the capacity to simultaneously integrate several different proteins, which may be useful for improving the efficiency of supra-molecular complex generation as well as producing multi-epitope particulate material suitable for vaccination.

## Results

### Detection of muNS domains implicated in inter-monomer interactions

**Figure 1:**
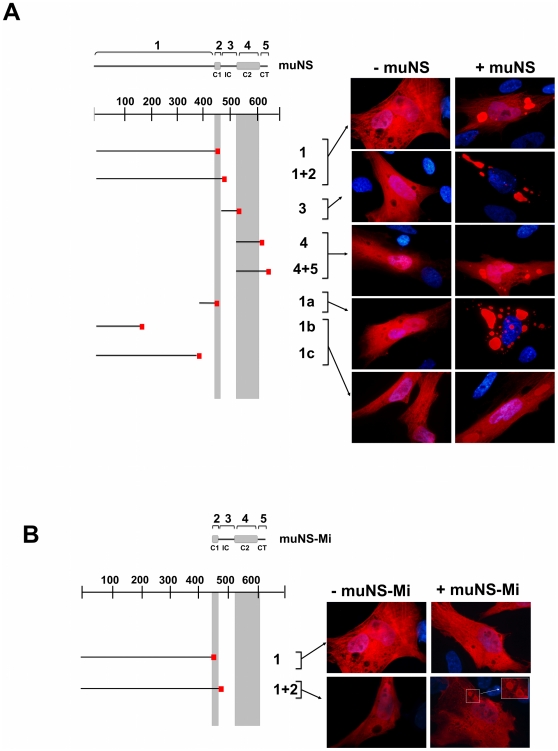
Incorporation of HA-tagged muNS regions into muNS or muNS-Mi-derived inclusions in transfected cells. **A**. muNS inclusions. Full-length muNS is schematically indicated by a horizontal black bar comprising residues 1–635 and regions 1 to 5 are also indicated. Horizontal black bars represent each single muNS fragment generated, with the HA epitope indicated as a small red box. The positions of two previously described coiled-coil elements predicted in the muNS sequence are indicated by two grey boxes and by vertical grey bars. Each construct was expressed alone (−muNS) or co-expressed with muNS (+muNS), and representative immunofluorescence images of transfected CEF cells are shown at the right side of the Figure. The HA epitope was detected by immunofluorescence (red) and nuclei were stained blue with DAPI. **B**. muNS-Mi inclusions. As in A, but the indicated constructs were expressed alone (−muNS-Mi) or co-expressed with muNS-Mi (+muNS-Mi). In the 1+2 image, the inset is an enlargement of the boxed area.

Avian reovirus muNS possesses several domains that are directly involved in self-association [11]. As a first approach to determine the role that the different muNS domains play in forming inter-monomer contacts, we decided to express individual muNS fragments and check their incorporation into muNS- and muNS-Mi-derived inclusions. We divided the protein muNS in 5 regions or domains (Figure 1A): the N terminal two-thirds of the protein that were shown to be dispensable to form inclusions but were involved in inclusion maturation [11] (domain 1, residues 1 to 447); and the four domains of muNS-Mi that were also previously described (C1 or domain 2, IC or domain 3, C2 or domain 4 and CT or domain 5, [11]). We constructed plasmids expressing the domains independently with a C-terminal hemagglutinin epitope tag. This tag allows us to differentiate the inclusions formed by full-length muNS and muNS-Mi from the HA-containing fragments by immunostaining. All constructs were sequenced and their expression checked by Western blot (not shown). For unknown reasons, we could not detect the expression of domains 2 and 5, either untagged, or HA-tagged at their N or C-terminus. To test their activity, we decided to add 2 and 5 to domains 1 and 4 respectively, to check the influence that their addition has on the incorporation of domains 1 and 4 into muNS inclusions (Figure 1A).

When individually expressed in CEF, none of the fragments shown in Figure 1 was able to form inclusions, but were evenly distributed throughout the cell (Figure 1A, left panels, -muNS). When co-expressed with full-length muNS the following results were obtained: i) domain 3 was exclusively detected in association with muNS inclusions, and is therefore the one that is recruited to inclusions with the best efficiency (Figure 1A, right panels, 3); ii) domain 1 also gets incorporated into inclusions quite efficiently, although a fraction of this protein was also detected in the nucleus and cytoplasm (Figure 1A, right panels, 1); iii) domain 4 showed some incorporation, although less than domains 1 or 3 (Figure 1A, right panels, 4); and iv) fusing 2 and 5 to domains 1 and 4 respectively, did not improve the incorporation efficiency of the latter domains. Furthermore, moving the HA tag to the N-terminus of domains 1 and 4 to avoid any interference with domains 2 and 5 had no effect on the incorporation of the fused constructs (not shown). Taken together, these results suggest either that 2 and 5 do not play an important role in muNS inter-monomer contacts, or that the approach used here is not suitable to uncover their roles. This situation contrasts with our previous observations that: i) domain 2 is directly implicated in establishing muNS inter-monomer contacts and ii) that domain 5 plays also a crucial role in inclusion construction [11]. Probably these two domains require additional muNS sequences for proper folding, proper spatial disposition, or both.

On the other hand, in our previous characterization of protein muNS [11], we have shown that domains 3 and 4 also play main roles in the inclusion formation, and that sequences of domain 1 are involved in inclusion maturation, but this is the first time where direct inter-monomer interaction is demonstrated for these three domains.

To map with more detail the domain 1-interacting sequences we expressed different fragments of this domain (1a, 381–448; 1b, 1–154; and 1c, 1–380) and analyzed their capacity to incorporate into muNS inclusions. Fragment 1a showed a good incorporating activity, similar to that of domain 3, whereas that of fragments 1b and 1c was very low (Figure 1A, right panels, 1a, 1b/1c). These results indicate that domain 1a is directly and strongly implicated in inter-monomer interactions. Remarkably, fragment 1a showed better incorporation efficiency than domain 1.

Domains 3, 4 and 5 showed similar incorporating activity when coexpresed with muNS-Mi instead of muNS (not shown), whereas domain 1 did not associate to muNS-Mi inclusions, suggesting that it interacts with sequences within the muNS region 1–448, upstream of muNS-Mi (Figure 1B, right panel 1). Addition of domain 2 to domain 1 did slightly improve the incorporation ability of domain 1, suggesting that sequences within domain 2 are involved in inter-monomer interactions (Figure 1B, right panel 1+2). With the full-length protein such improvement could not be detected, probably masked by the better incorporation of domain 1 itself.

### Using muNS domains as molecular tags for cytoplasmic inclusion targeting

**Figure 2:**
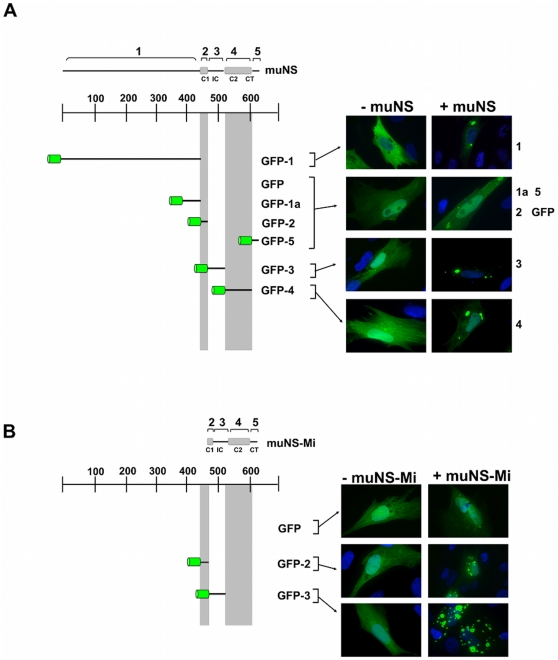
Incorporation of GFP tagged with muNS regions into muNS or muNS-Mi-derived inclusions in transfected cells. **A**. muNS inclusions. Full-length muNS and GFP-fused muNS regions are represented as in Figure 1, with the green fluorescent protein represented as a green barrel. Each construct was expressed alone (−muNS) or co-expressed with muNS (+muNS), and representative fluorescence images of transfected CEF cells are represented at the right side of the Figure. Nuclei were stained blue with DAPI. **B**. muNS-Mi inclusions. As in A, but the indicated constructs were expressed alone (−muNS-Mi) or co-expressed with muNS-Mi (+muNS-Mi).

The results of the experiments shown in Figure 1 prompted us to use muNS domains as molecular tags for targeting proteins of interest to muNS-derived inclusions, which would constitute a novel tagging system with many potential applications. To explore this possibility, we tagged the green fluorescent protein (GFP) with different muNS domains, and analyzed its incorporation into muNS and muNS-Mi inclusions. GFP was chosen for this assay because it can be detected by fluorescence without antibodies, and also because its auto-fluorescence capability relies on its correct folding, thus allowing us to easily monitor the proper folding of the inclusion-associated tagged GFP. Although we were unable to detect the expression of untagged and HA-tagged domains 2 and 5, we could successfully use them to tag GFP (Figure 2A). Thus, all five domains described in Figure 1, as well as subdomain 1a, were used for the tagging experiment described in Figure 2. All recombinant plasmids expressing the GFP chimeras shown in Figure 2 were sequenced and their protein expression checked by Western blot (not shown). Like GFP, all the chimeras were evenly distributed throughout the cell when expressed alone (Figure 2A, left panels, -muNS). However, when co-expressed with muNS the following results were obtained (Figure 2A): i) untagged GFP distributed uniformly throughout the whole cell including the nucleus and, although it was not excluded from muNS inclusions, it associated with them very poorly (Figure 2A, right panels, GFP); ii) domain 1, but not subdomain 1a, successfully directed the GFP moiety to inclusions, suggesting that the attached GFP somehow hinders 1a interaction with muNS monomers (Figure 2A, right panels 1 and 1a); iii) GFP tagged with domains 2 and 5 behaves exactly the same as untagged GFP (Figure 2A, right panels, 1a, GFP); iv) domain 4 efficiently targeted tagged GFP to inclusions, although a minor fraction of this protein was diffusely detected in the cytoplasm and nucleus (Figure 2A, right panels, 4); and v) domain 3 is the best tagging domain as 3-tagged GFP was exclusively detected within inclusions (Figure 2A, right panels, 3). When muNS-Mi was used as inclusion-forming unit (Figure 2B), the same results were obtained except that: i) GFP fused to domain 1 did not incorporate into muNS-Mi inclusions (not shown); and ii) GFP-domain 2 (GFP-2), which did not incorporate into muNS inclusions, showed poor but significant incorporation into muNS-Mi inclusions.

### IC-tagging for protein purification

Some of the tested muNS domains were shown to be useful tags for targeting GFP to muNS inclusions. Domain 3, that we have previously named Intercoil (IC), seems the most adequate for such purpose, since it is very small and very efficient in directing a tagged protein to the inclusions formed by muNS and muNS-Mi. Domain 1 also works quite efficiently with full-length muNS, but not with muNS-Mi, and besides it is too large for a suitable tag. Although domain 4 has a small size and works with both inclusion-forming units, it is not as efficient as IC.

**Figure 3:**
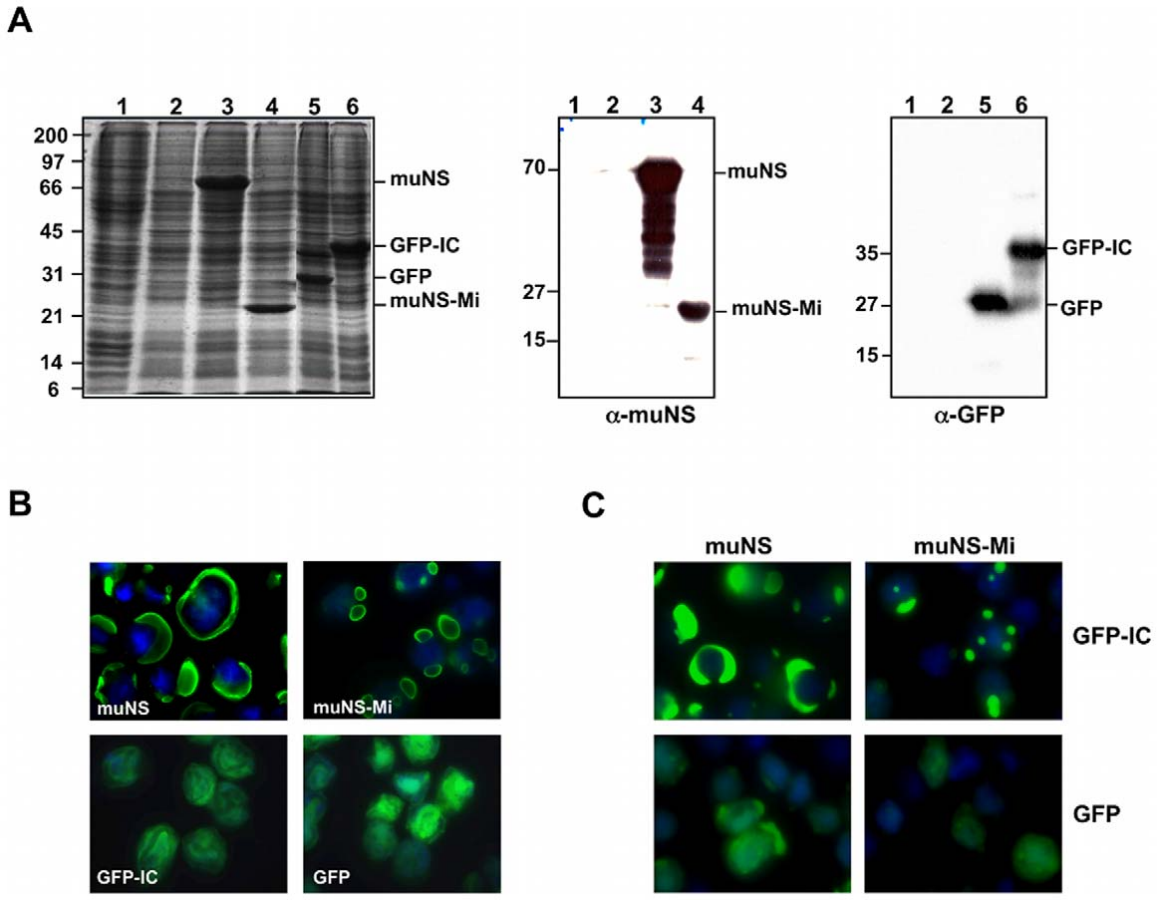
Incorporation of GFP-IC into muNS-derived inclusions in baculovirus-infected cells. **A**. SDS-PAGE and immunoblot analysis. Extracts from Sf9 cells were subjected to SDS-PAGE analysis and the gel was stained with Coomassie blue (left panel). Extracts from mock-infected and wild-type baculovirus-infected Sf9 cells are shown in lanes 1 and 2 respectively. Lanes 3 to 6 show extracts from Sf9 cells infected with recombinant baculovirus expressing muNS (lane 3), muNS-Mi (lane 4), GFP (lane 5) and GFP-IC (lane 6). The extracts were also subjected to Western blot analysis with anti-muNS antibodies (middle panel) or with an anti-GFP monoclonal antibody (right panel). The positions of the recombinant proteins are indicated on the right and the molecular weight markers on the left. **B–C**. Immunofluorecence analysis. Sf9 cells were infected with recombinant baculoviruses expressing muNS, muNS-Mi, GFP-IC and GFP (panel B), or co-infected with recombinant baculoviruses expressing muNS (left column) or muNS-Mi (right column) and GFP-IC (upper row) or GFP (lower row) (panel C). After 72 h, the cells were fixed and immunostained with rabbit antibodies against muNS (green), except those containing GFP that were directly detected. Nuclei were stained blue with DAPI.

We have recently devised a simple protocol for purifying the inclusions generated by baculovirus expression of muNS and muNS-Mi in insect cells [11]. Thus, we decided to use this protocol for purifying inclusion-associated IC-tagged proteins expressed in baculovirus-infected cells. For this, recombinant baculoviruses expressing untagged and IC-tagged GFP were constructed, and the latter was engineered to contain a protease factor Xa target sequence between IC and GFP, in order to facilitate GFP release and subsequent purification. Analysis by SDS-PAGE and Coomassie-blue staining of extracts from cells infected with these baculoviruses revealed the presence of two prominent bands with the electrophoretic mobility expected for both GFP (Figure 3A, lane 5) and GFP-IC (lane 6, GFP-IC), which were not present in uninfected or wild-type baculovirus-infected cells (lanes 1 and 2). The identity of the two proteins was confirmed by Western blot using monoclonal antibodies against GFP (Figure 3A, right panel). The expression of muNS (lane 3) and muNS-Mi (lane 4) is also included in the stained gel. Analysis of the expressed proteins by fluorescence microscopy revealed that while GFP and GFP-IC distributed diffusely throughout the whole cell when individually expressed, muNS and muNS-Mi accumulated into large cytoplasmic inclusions (Figure 3B). However, GFP-IC, but not GFP, relocated to inclusions in cells coexpressing either muNS or muNS-Mi (Figure 3C). These results showed both that the incorporation of GFP into inclusions does not dismantle the inclusions and that GFP is properly folded while inclusion-associated, because it still emits its characteristic fluorescence. These results further indicate that the tagging and relocalization system described here works independently of the cell type and expression system.

**Figure 4:**
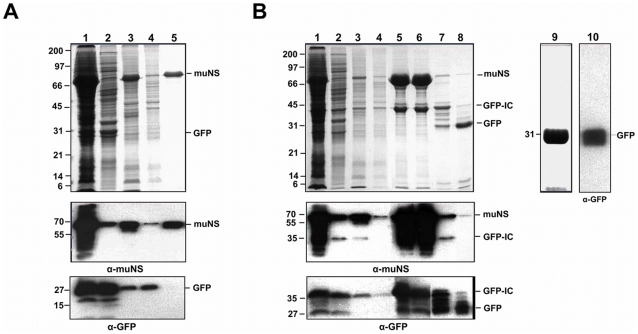
Purification of GFP targeted to muNS inclusions. **A**. Untagged GFP. Insect Sf9 cells co-infected with recombinant baculoviruses expressing muNS and GFP were lysed in hypotonic buffer at 72 h.p.i., and the resulting cell extract (lane 1) was fractionated by centrifugation into pellet and supernatant fractions (the supernatant fraction is shown in lane 2). The pellet was then washed twice with hypotonic buffer, resuspended in the same volume of hypotonic buffer and sonicated. The sonicated extract (lane 3) was centrifuged (the supernatant fraction is shown in lane 4), and the pelleted inclusions were washed five times with hypotonic buffer (lane 5). All samples were analyzed by 12% SDS-PAGE and the protein bands were visualized by Coomassie blue staining (upper panel). The samples were also subjected to Western blot analysis with anti-muNS antibodies (middle panel) or with anti-GFP monoclonal antibodies (bottom panel). The positions of muNS and GFP are indicated on the right and that of the molecular weight markers on the left. **B**. IC-tagged GFP. Protein expression and inclusion purification were performed as above. The final purified pellet was resuspended in 500 mM of NaCl (lane 5) and centrifuged. The supernatant (lane 6) was loaded on a desalting column. The eluted material was centrifugated again and the supernatant (lane 7) was incubated with factor Xa (lane 8). Free GFP was purified by ion exchange chromatography and the GFP-containing fractions were pooled and concentrated (lane 9) and subjected to Western blot analysis with an anti-GFP monoclonal antibody) (lane 10). Samples 1–8 were subjected to Western blot analysis with antibodies against muNS (middle panel) or GFP (bottom panel). The positions of muNS and GFP-IC proteins are indicated on the right and that of the molecular weight markers on the left.

The inclusions made by muNS in insect cells coexpressing GFP or GFP-IC were purified as described in the legend for Figure 4. After cell lysis in hypotonic buffer and subsequent centrifugation, most GFP-IC (Figure 4B), but not GFP (Figure 4A), remained associated to pelleted muNS inclusions, indicating that the association of GFP-IC with muNS inclusions is promoted by the Intercoil tag. Furthermore, the association of GFP-IC to muNS inclusions was not disrupted during the purification process, as revealed by its presence in the final purified inclusions (Figure 4B, lane 5). It should be pointed out that the low protein amount observed in lane 3 of Figure 4B is caused by the inability of pelleted inclusions to be resuspended in the absence of salt, since higher protein amounts were detected when salt was used for resuspending the final pelleted inclusions (lane 5). Salt was not used in this step because it would dismantle the inclusions and abort GFP purification. After dismantling the final purified inclusions with salt, the sample was centrifuged and the supernatant shown in lane 6 was desalted and centrifuged again. The resulting supernatant contained negligible amounts of muNS (lane 7), which were eliminated upon storage in low-salt buffer (not shown). The purified GFP could be used at this stage, without the need of an affinity column to purify the soluble tagged protein. Although lane 7 shows some bands between the positions of tagged and untagged GFP, the Western blot in the lower panel demonstrates that all are cleavage fragments of GFP-IC. We have observed that the IC tag becomes quite labile after inclusion solubilization. To further purify the protein from the IC tag we followed the standard methods used in any other affinity purification method. Thus, incubation of the final supernatant with factor Xa released free GFP (lane 8), which was subsequently separated from IC and factor Xa by ion-exchange chromatography. The GFP-containing chromatographic fractions were pooled and concentrated, and the analysis of the final sample by SDS-PAGE (Figure 4B, lane 9) and by Western blot (Figure 4B, lane 10) showed that it contains pure GFP. The same results were obtained when using muNS-Mi as the inclusion-forming unit (not shown), instead of muNS.

In order to increase the versatility of our method, two new recombinant baculoviruses expressing GFP-muNS and GFP-muNS-Mi were generated. The chimeric proteins not only generated inclusions that could be easily purified (not shown), but formed fluorescent green inclusions, which greatly facilitates their detection and monitoring during the purification process. This method represents a novel, inexpensive and simple approach for the purification of proteins expressed in baculovirus-infected cells.

### Inclusion-immobilization of active enzymes

The fact that avian reovirus replication and morphogenesis takes place exclusively within viral factories indicates that the viral enzymes involved in these processes are able to display their catalytic activity while inserted into these structures. In the same way, IC-tagged enzymes might retain their activity when incorporated into muNS-derived inclusions and, if this is true, the enzymatic activity could be easily removed from the solution, after completion of the reaction time by a simple centrifugation step. This would be useful for eliminating enzymes from processes where serial reactions are needed and/or for reusing the enzyme in another reaction.

**Figure 5:**
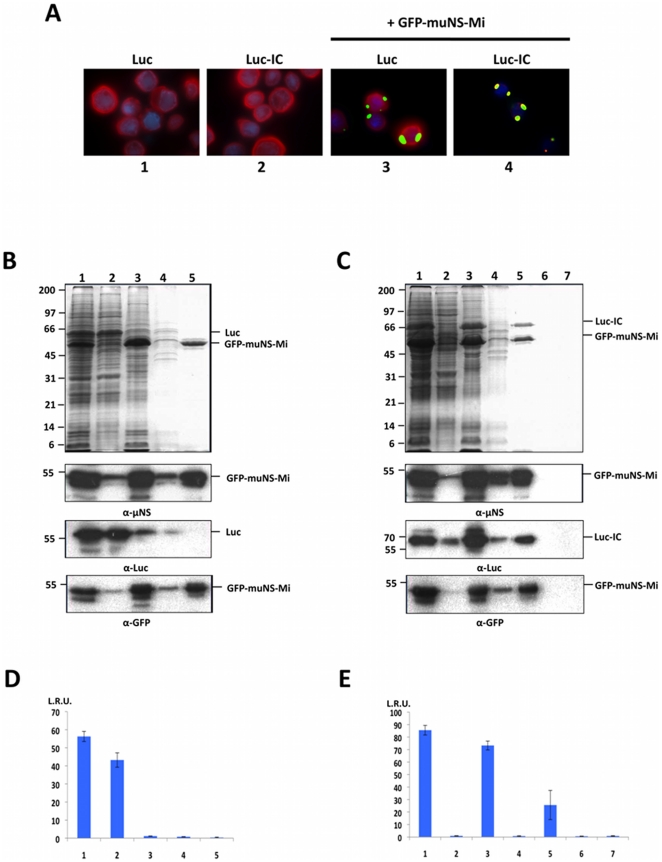
Purification of Luciferase targeted to GFP-muNS-Mi inclusions. **A**. Recruitment of Luc-IC to GFP-muNS-Mi inclusions in Sf9 cells. Sf9 cells were co-infected with the recombinant baculoviruses expressing the proteins shown on top of the pictures. The cells were then fixed and immunostained with anti-Luc antibodies (red), and the constructs containing GFP were visualized without antibodies (green). Nuclei were stained with DAPI (blue). **B**. Untagged Luc. The same purification steps shown in Figure 4 for muNS and GFP are here shown for GFP-muNS-Mi and Luc. Samples of each purification step were also subjected to Western blot analysis with anti-muNS antibodies, with anti-Luc antibodies or with anti-GFP monoclonal antibody as indicated in the Figure. The positions of GFP-muNS-Mi and Luc proteins are indicated on the right and the molecular weight markers on the left. **C**. IC-tagged Luc. Lanes 1 to 5: as in B, but using IC-tagged Luc (Luc-IC) instead of the untagged Luc. The sample shown in lane 5 was centrifuged (lane 6) or filtered through a 0.22 µM filter (lane 7). All samples were resolved by 12% SDS-PAGE and protein bands were visualized by Coomassie blue staining. Samples of each purification step were subjected to Western blot as indicated above. The positions of GFP-muNS-Mi and Luc-IC proteins are indicated on the right and the molecular weight markers on the left. **D–E**. Determination of Luciferase activity of the samples shown in B and C respectively. Bars represent Luc levels present in samples of each purification step. R.L.U. are indicated on the left. The error bars indicate standard deviations.

*Photinus pyralis* firefly Luciferase (Luc) was used to test the utility of our method for purifying active enzymes. For this, recombinant baculoviruses expressing Luc and IC-tagged Luc (Luc-IC) were generated and used for infecting insect cells. Immunofluorescence analysis of the infected insect cells revealed that both luciferase proteins were diffusely distributed in the cytoplasm (Figure 5A, panels 1 and 2). Untagged and tagged Luc displayed similar specific activity (not shown), indicating that IC tagging has no negative effect on Luc activity. As with GFP, IC tagging caused relocation of Luc to muNS-related inclusions in insect cells co-expressing GFP-muNS-Mi (Figure 5A, panel 4), while untagged Luc showed no association with inclusions (Figure 5A, panel 3). Similar results were obtained when using muNS, muNS-Mi and GFP-muNS as inclusion-forming units, instead of GFP-muNS-Mi. Next, Luc-containing inclusions were purified using the same protocol shown in Figure 4, and the final purified inclusions were shown to contain GFP-muNS-Mi and Luc-IC (Figure 5C, lane 5), but not untagged Luc (Figure 5B, lane 5). Furthermore, a similar value of relative activity was obtained when we performed densitometric analysis of inclusion-free and inclusion-associated Luc, demonstrating that its association with muNS structures does not negatively affect its activity (not shown). Unlike the inclusions containing GFP-IC and muNS (Figure 4B), the pelleted inclusions containing GFP-muNS-Mi Luc-IC could be easily resuspended without the use of salt, suggesting that the solubility of the inclusions is influenced by the nature of the inclusion-forming unit and/or the tagged protein. Thus, having four different inclusion-forming units makes our system more adaptable for protein purification.

The Luc activity of the extracts shown in the stained gels of Figures 5B and 5C were measured. The results shown in Figures 5D and 5E, respectively, confirmed that untagged Luc is lost in the initial supernatants (Figure 5D, lane 2), since no activity is detected in the final purified pellet (Figure 5D, lane 5). However, Luc-IC not only remains strongly associated to inclusions (Figure 5C, lane 5), but also retains its enzymatic activity (Figure 5E, lanes 3 and 5). The inclusions could be easily removed from solution in one simple step, either by centrifugation (Figure 5C, lane 6) or filtration through a 0.22 µm membrane (Figure 5C, lane 7). In both cases, the Luc activity is completely removed (Figure 5E, lanes 6 and 7).

**Figure 6:**
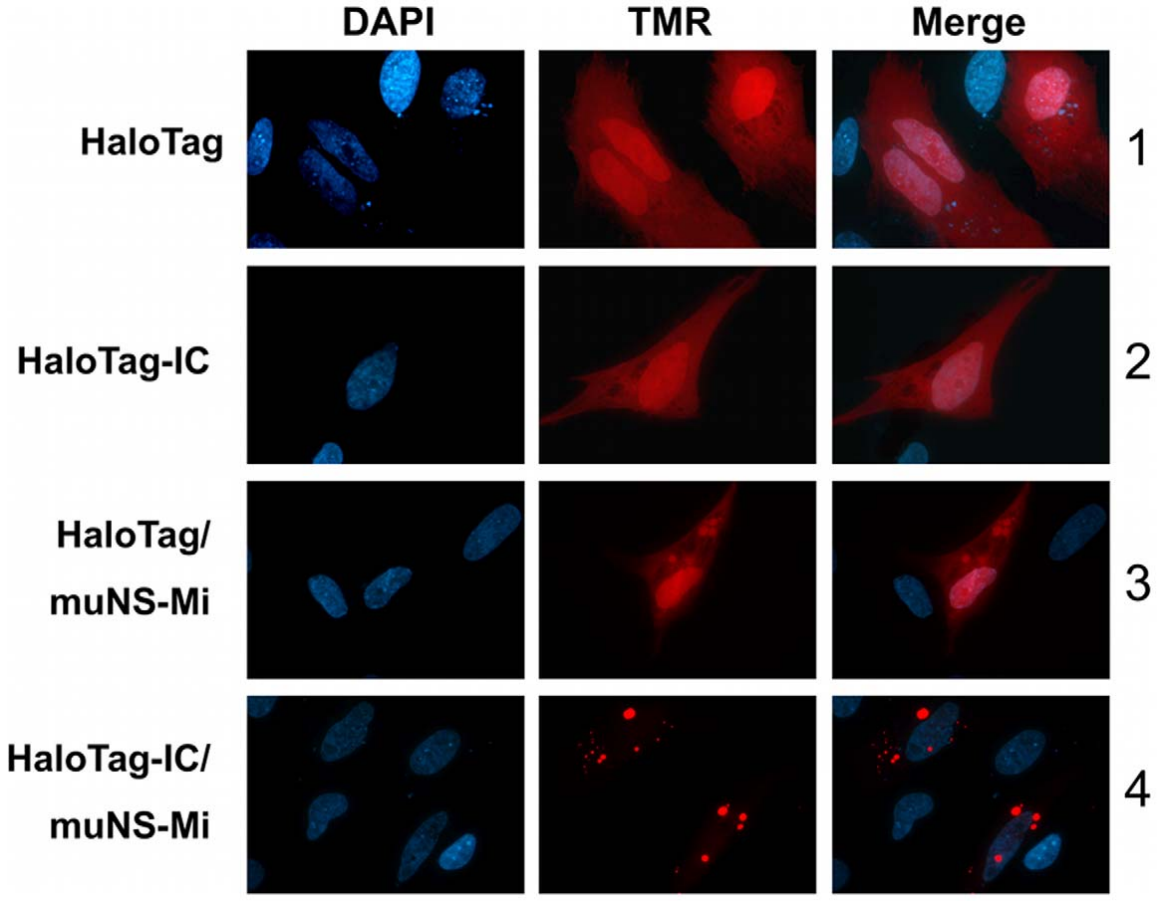
Incorporation of HaloTag-IC into muNS-Mi inclusions in transfected cells. Monolayers of CEF were transfected with the plasmids expressing the proteins indicated on the left of the figure. After 24 h incubation at 37°C, the cells were incubated with the TMR ligand (red) and subsequently fixed and visualized with a fluorescence microscope. Nuclei were stained blue with DAPI.

Since Luc activity of purified inclusions had been analyzed in vitro, we tried to determine whether the activity of an inclusion-associated IC-tagged protein could also be monitored in vivo. For this, we used the HaloTag protein (Promega Corp.), which is a genetically modified hydrolase that is able to catalyze its covalent binding to a series of membrane-spanning ligands and can be used for in vivo labeling [15]. Thus, HaloTag and IC-tagged HaloTag (HaloTag-IC) were transiently expressed in transfected CEF cells. The transfected cells were then labeled in vivo with tetramethyl rodamine (TMR) ligand and the HaloTag intracellular distribution was analyzed by fluorescence microscopy. Both proteins were diffusely distributed throughout the whole cell and bound TMR, showing that IC tagging did not affect HaloTag activity (Figure 6, rows 1 and 2). When the HaloTag proteins were co-expressed with muNS-Mi, we did not observe any changes in the untagged HaloTag distribution (Figure 6, row 3) but, as expected, HaloTag-IC was completely relocated to inclusions (Figure 6, row 4). In addition, both proteins showed the same TMR labeling efficiency, demonstrating that inclusion association does not diminish HaloTag activity. Similar results were obtained with all four described inclusion-forming proteins (not shown).

### Simultaneous targeting of several proteins to muNS-related inclusions

**Figure 7:**
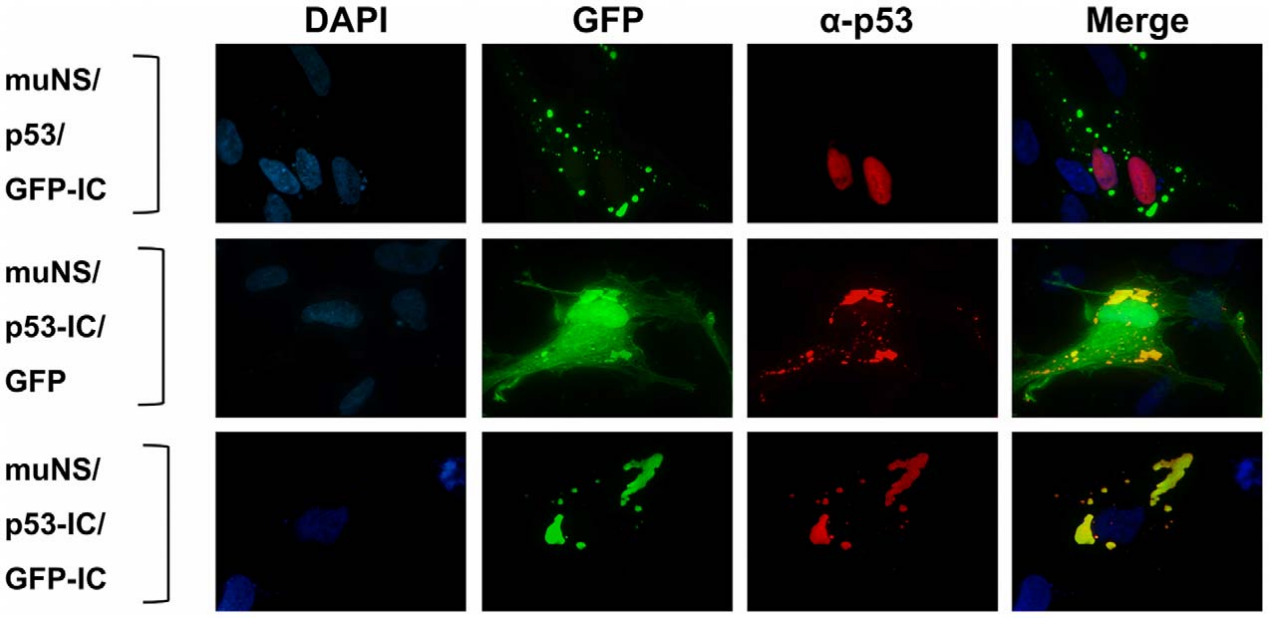
Simultaneous targeting of GFP-IC and P53-IC to muNS inclusions. Semiconfluent monolayers of CEF were transfected with the plasmids expressing the proteins indicated on the left of the Figure. The cells were immunostained with an anti-p53 monoclonal antibody (red) and GFP was directly visualized (green). Nuclei were stained with DAPI (blue).

The versatility of our inclusion-targeting system would be greatly improved if several proteins could be recruited simultaneously to muNS-derived inclusions. To test this possibility, we used two different IC-tagged proteins, GFP-IC [11] and p53-IC. Instead of baculovirus-infected insect cells we used transfected CEF cells where individual inclusions are dispersed throughout the cytoplasm making easier to monitor by immunofluorescence the integration of individual proteins in the same inclusion. We first demonstrated that both untagged p53 and GFP do not associate with muNS-inclusions (not shown). We also checked that p53 does not incorporate into GFP-IC-containing muNS inclusions (Figure 7, upper row), and that GFP does not integrate into p53-IC-containing inclusions (Figure 7, middle row). Strikingly, we observed that p53-IC was completely relocated to cytoplasmic inclusions when coexpressed with muNS (Figure 7, middle row), indicating that our inclusion-targeting system can also be used for recruiting a nuclear protein like p53. Finally, when monolayers of CEF cells were cotransfected with plasmids expressing GFP-IC, p53-IC and muNS, the inclusions formed by muNS were found to contain both IC-tagged proteins, indicating that our inclusion- targeting system enables the simultaneous integration of more than one protein into muNS inclusions.

## Discussion

We have previously investigated the inclusion-forming contribution of the different domains of the avian reovirus non-structural protein muNS, by expressing N- and C-terminal muNS truncations and by replacing different muNS domains by dimeric proteins [11]. We determined that the N-terminal two thirds of the protein are dispensable for inclusion formation but in some way regulates or influences the shape of the inclusions formed by muNS. On the other hand, we showed that the muNS C-terminal one third contains 4 different domains that are absolutely essential for inclusion formation. We were able to show that is possible to replace domain 2 by different dimeric domains, and that domain 5 determinates the inclusion formation efficiency and inclusion shape. However, in that study we could not determine how these domains interact with each other to create the highly structured muNS inclusions.

In order to gather more information about muNS sequences involved in intermonomer contacts, we decided to try alternative approaches. First of all, we tried the mammalian two-hybrid system for analyzing the interaction of different muNS regions with muNS and with muNS truncations. However, the results were not satisfactory, since we were not able to detect the expression of several constructs, and some others were expressed in aggregated form. As an alternative, we developed the inclusion-targeting protocol described in the results section that allowed us to reach some conclusions that are novel with respect to the muNS inclusion construction mechanisms. Thus, this is the first time that is shown a direct interaction of both coiled-coil domains in muNS (domains 2 and 4) with regions within muNS-Mi. Taking into account that domain 2 can be replaced by dimeric domains [11] and that coiled-coils are frequently involved in homo-oligomeric contacts, our results suggest that both coiled-coil elements make homo-domain contacts with identical domains from different muNS monomers.

Strikingly, our inclusion-targeting strategy failed to reveal the presence of interacting sequences within domain 5, in spite that this domain has been previously shown to be an essential inclusion-forming player by orientating monomer-to-monomer interactions [11]. It is possible that domain 5 does not exert its activity by interacting with muNS domains and/or it is not properly folded when expressed alone. On the other hand, our results revealed that domain 1a, which is a small domain 1 segment, incorporates into inclusion with better efficiency than domain 1. Strikingly, we were able to detect the incorporation to the inclusions of domain 1, but not of domain 1a when these domains were expressed as GFP-fusions. These results highlight the importance of using different strategies to detect interacting domains, since negative results obtained by using just one approach might be an artifact caused by limitations inherent to the approach, like steric hindrance or deficient folding, especially when expressing fragments or fusions and not individual full-length proteins.

From the positive results obtained in this study with domain 1, we can deduce that there are sequences within the region 381-448 that are directly involved in inter-monomer interactions. Such sequences have not been previously described and probably form part of the muNS region that we have previously shown to influence inclusion size and morphology [11]. Our results further suggest that interacting sequences within domain 1 make contacts with sequences within the same domain of another muNS monomer, since domain 1 incorporates into the inclusions formed by muNS, but not by domain 1-lacking muNS-Mi.

Domain 3 or IC produced the best inclusion-targeting results, incorporating into muNS and muNS-Mi inclusions in a very efficient way. Previous studies performed with both avian and the related mammalian reoviruses [11], [16] had shown that the Intercoil domain is very important in the muNS inclusion construction, because point mutations of two critical His and Cys residues abolished all the muNS ability to form inclusions. Some authors had reasoned that they could represent half of a metal-chelating domain and that the full chelating domain would be formed by muNS-muNS dimerization or by dimerizing with a different protein [16]. However, this is the first time where direct and strong interaction ability is demonstrated for the IC domain, something that adds some support to the mentioned theory. The reproducibility of the results obtained with the IC suggests that it represents a domain with a very independent folding. The strong interaction with the inclusions, the reproducibility of its results and its small size prompted us to use it as a protein tag. In our hands it worked perfectly, promoting inclusion incorporation without modifying the activity and/or intracellular distribution (in absence of the inclusion) of the tagged protein.

We have recently developed a simple protocol for purifying the inclusions formed by baculovirus-expressed muNS in insect cells [11]. Here, we have adapted that protocol to the purification of IC-tagged proteins, and have successfully used that protocol for purifying IC-tagged GFP and luciferase in a simple an inexpensive way. In both cases the purified proteins remained active throughout the purification process. The final purified product can be soluble or inclusion-integrated. Inclusion-integrated proteins remain active and can be utilized to catalyze enzymatic reactions. Immobilized enzymes can be easily removed from solution for purification of the product and/or allow enzyme re-utilization. Thus, our system integrates purification and enzyme-immobilization, which is an efficient process widely used in industry. Additionally, the use of HaloTag also allowed us to establish that the inclusion-immobilized proteins are also active in vivo, something that could be useful for different applications. Our tagging and inclusion-targeting system described in the present study was very reliable, since it worked well for all the proteins tested by us. The system allowed inclusion targeting of nuclear and cytoplasmic proteins, expressed in baculovirus-infected, or avian transfected cells.

We have detected that the nature of the tagged protein can sometimes influence the texture of the containing inclusions, producing sticky pellets that are not easily re-suspended. That circumstance can be circumvented in our system by making use of the four different inclusion-forming proteins, which present slightly different characteristics between them: muNS, muNS-Mi, GFP-muNS and GFP-muNS-Mi. As an example, the Luciferase purification results shown in Figure 5 were obtained with inclusions formed by GFP-muNS-Mi, while Luc-IC integrated into GFP-muNS inclusions produced pellets that were more difficult to handle. Furthermore, the use of GFP-fused inclusion-forming proteins facilitates the purification process, since they allow following the destiny of the inclusions along the purification process by its green color and/or fluorescence, which adds more a versatility and adaptability to our purification system.

A nuclear protein like p53 is completely relocalized to the cytoplasmic muNS-derived inclusions when IC-tagged. This represents a novel way to purify nuclear proteins in eukaryotes without having to use a different protocol for their solubilization and extraction from the nucleus, which represents an additional advantage over pre-existing purification methods.

Avian reoviruses use viral factories to sequentially concentrate the viral components required for viral replication and morphogenesis [7]. For mammalian reoviruses, it was established that different regions of their muNS protein attract different structural capsid proteins without interfering with the stoichiometry of capsid assembly [17]. In a similar way, our inclusion-targeting system, which has been proved to be suitable for the simultaneous recruitment of several proteins, should be expected to increase the building efficiency of supra-molecular complexes, a step that is currently the bottleneck for many structural and functional studies. Although further studies are required to demonstrate the capability of our method for the generation of supramoleuclar complexes, our preliminary results indicate that the DsRed protein, whose tetramerization is essential for its autofluorescence [18], is still fluorescent when IC-tagged and integrated into muNS-derived inclusions (not shown), which in turn indicates that IC-tagging and inclusion-targeting does not avoid the proper interaction between the tagged proteins. To increase the versatility of this approach, we are now testing the capacity of other muNS domains (2, 4 and 1a) to function as molecular tags for directing proteins to inclusions, which would allow directing proteins containing different tags to the same inclusion.

Our system might be also exploited to produce immunogens that would have potential advantages as vaccines: i) inclusions are particulate matter, and particulate immunogens are the best for stimulating both humoral and cellular immune responses [19]; ii) inclusion-derived immunogens can be easily produced and purified; iii) they should be biologically safe, because organisms would be immunized with proteins and not with genetic material or viruses; and iv) different epitopes can be simultaneously exposed on the same particle. In that way, for example we could arrange several epitopes of a single virus, or epitopes from different serotypes on the same vaccine to increase its overall efficiency.

The results presented here shed light on the general rules that govern the construction of the highly structured protein aggregates formed by protein muNS and additionally allowed to develop a novel protein tagging and inclusion-targeting system with many potential applications.

## Materials and Methods

### Cells, viruses and antibodies

Primary cultures of chicken embryo fibroblasts (CEF) were prepared from 9- to 10-day-old chicken embryos [20] and grown in monolayers in medium 199 supplemented with 10% (w/v) tryptose-phosphate broth and 5% (v/v) calf serum. The Sf9 insect cell line was grown in suspension culture at 27°C in serum-free Sf-900 II medium (Invitrogen, Barcelona, Spain). Propagation of baculoviruses in Sf9 cells has been described previously [21]. Rabbit polyclonal serum against the avian reovirus S1133 muNS protein was raised in our laboratory [10]. Goat polyclonal antibody specific for recombinant firefly Luciferase (*Photinus pyralis*) was from Promega (Madrid, Spain). Mouse monoclonal antibody against *Aequorea victoria* green fluorescent protein (GFP) was from Roche (Barcelona, Spain). Rabbit polyclonal antibodies against the influenza virus hemagglutinin (HA) epitope, monoclonal antibody PAB40 specific for p53, Cy3 conjugated antibodies against both goat and rabbit IgG, and Alexa 594 conjugated antibody against mouse IgG, all were from Sigma-Aldrich (Madrid, Spain).

### Transfections, IF microscopy and labeling with TMR Ligand

Transfections of preconfluent cell monolayers were performed with the Lipofectamine Plus reagent (Invitrogen, Barcelona, Spain) according to the manufacturer's instructions. Transfected cells were incubated at 37°C for 24 h, unless otherwise stated.

For tetramethyl rodamine (TMR) labeling, transfected cells were incubated for 15 min with TMR, then the cells were rinsed twice with PBS and incubated in fresh medium for 30 min. The medium was removed, and the cells were washed 3 times with PBS before being fixed with 4% paraformaldehyde and used for imaging. Images were obtained with an Olympus DP-71 digital camera mounted on an Olympus BX51 fluorescence microscope, and processed with Adobe Photoshop (Adobe Systems, USA). For proper image acquisition and better resolution of the inclusions, images of muNS-derived inclusions or inclusion-containing proteins are underexposed in comparison to those of cells lacking inclusions. For indirect immunofluorescence, cell monolayers grown on coverslips were infected or transfected as indicated in the Figure legends and, at the indicated times, the monolayers were washed twice with PBS and fixed with 4% paraformaldehyde in PBS at room temperature for 10 min. Paraformaldehyde-fixed cells were washed twice with PBS, incubated for 4 min in permeabilizing buffer (0.5% Triton X-100 in PBS), and then incubated for 1 h at room temperature with primary antibodies diluted in blocking buffer (2% bovine serum albumin in PBS). The cells were then washed three more times with PBS and incubated with secondary antibodies and with 1 µg/ml of DAPI (4′, 6′-diamidino-2-phenylindole)/ml. The coverslips were then washed six times with PBS and mounted on glass slides. Images were obtained and processed as indicated above.

### Immunoblotting

For Western blot analysis, cell extracts were resolved by SDS-PAGE and proteins in unfixed gels were transferred to PVDF membranes (Immobilon-P Millipore, Madrid, Spain) for 1 h at 100 mA in a semidry blotting apparatus (Bio-Rad, California, USA). Protein bands were detected with specific antibodies using the Immobilon Western Chemiluminiscent HRP Substrate (Millipore, Madrid, Spain).

### Plasmid constructions

Plasmid pCMV-p53, expressing the human p53 protein was a generous gift of Dr. Anxo Vidal (Universidad de Santiago de Compostela) and has been previously described [22]. The construction of pCINeo-muNS, which expresses full-length muNS, and of pCINeo-muNS(448–635), which expresses muNS-Mi have been described [9], [11].

#### i) muNS-GFP fusions

The construction of most of the recombinant plasmids, which express GFP fused to the N termini of different muNS regions has been described previously [11].

To express GFP fused to the N terminus of the muNS(381–448) (this construction was named GFP-1a in results), the recombinant plasmid pGEMT-M3 [9] was subjected to PCR amplification with the following primers. The forward primer was 5′-GCGGAATTCTATGCCATCCTTCTTACTCGGTG-3′(EcoRI site is single underlined) and the reverse primer was 5′-GCGGGATCCTTATGGACCAACGGACGAATCG-3′ (BamHI site is single underlined and the stop codon is double underlined). The resulting amplified product was digested with EcoRI and BamHI and then ligated to the pEGFP-C1 vector (Clontech, Saint Germain en Laye, Francia), that had been cut with the same enzymes, to generate the recombinant plasmid pEGFP-C1-M3(381-448).

To express GFP-IC (where the factor Xa cleavage site (Xacs) is fused to the C terminus of GFP and to the N terminus of muNS(477–542), the recombinant plasmid pGEMT-M3 [9] was subjected to PCR amplification with the following primers: the forward primer was 5′-GCGGAATTCTATCGAGGGAAGGGAAGATCACTTGTTGGCTTATC-3′ (EcoRI site is single underlined and factor Xa cleavage site is double underlined) and the reverse primer 5′-GCGGGATCCTTACGCTTCCACACGGGGTTCCCAC-3′ (BamHI site is single underlined and the stop codon is double underlined). The PCR product was cut with EcoRI and BamHI and ligated to pEGFP-C1 that had been cut with the same enzymes, to generate the recombinant plasmid pEGFP-C1-Xacs-muNS(477–542).

#### ii) HaloTag-IC

To express HaloTag-IC, the recombinant plasmid pGEMT-M3 [9] was subjected to PCR amplification with the following primers. The forward primer was 5′-GCGTCTAGAATCATGGCGGAAGATCACTTGTTGGCTTATC-3′ (XbaI site is single underlined) and the reverse primer was 5′-GCGGGGCCCTTACGCTTCCACACGGGGTTCCCAC-3′ (ApaI site is single underlined and the stop codon is double underlined). The resulting amplified product was digested with XbaI and ApaI and then ligated to the pCDNA3.1/Zeo vector (Promega, Madrid, Spain) that had been cut with the same enzymes, to generate the recombinant plasmid pCDNA3.1/Zeo-muNS(477–542). The HaloTag sequence was obtained by PCR-amplification using the plasmid pHT2 as template (Promega, Madrid, Spain), and the following primers: the forward primer was 5′-GCGGGATCC ACCATGGGCTCCGAAATCGGTACAGGC-3′ (BamHI site is single underlined and the start codon is double underlined), and the reverse primer was 5′- GCATAAGAATGCGGCCGCCAGCCGGCCAGCCCGGGGAG-3′ (NotI site is single underlined). The PCR product was digested and cloned into the BamHI and NotI sites of pCDNA3.1/Zeo-muNS(477–542) to generate pCDNA3.1/Zeo-HaloTag-muNS(477–542).

#### iii) p53-IC

To express p53-IC, the recombinant plasmid pCMV-wtp53 [22] was subjected to PCR amplification with the following primers: the forward primer was 5′-GCGGGATCCATCATGGAGGAGCCGCAGTCAGATCC-3′ (BamHI site is single underlined and the start codon is double underlined), and the reverse primer was 5′-GCGGAATTCGTCTGAGTCAGGCCCTTCTGTCTTG-3′ (EcoRI site is single underlined) to amplify the complete human p53 coding sequence. The PCR product was cut with BamHI and EcoRI and then ligated to pCDNA3.1/Zeo-muNS(477–542) that had been cut with the same enzymes.

#### iv) muNS-HA fusions

To express the influenza virus hemagglutinin epitope (HA) fused to the C-terminus of different muNS regions, the HA-encoding sequence, the start and stop codons and the restriction sites were introduced at different positions of the M3 gene by PCR amplification of the desired M3 region. Each PCR was performed using pGEMT-M3 as a template [9], and the primers used are listed in Table 1. Each PCR product was cut with EcoRI and XbaI and then ligated to the pCDNA3.1/Zeo vector that had been cut with these same enzymes. Each construct was checked by sequencing and Western blot analysis and named for the muNS residues that the expressed protein contains (Table 1). The correctness of the constructs was confirmed by sequencing and Western blot analysis of the expressed proteins.

**Table 1:** Construction of plasmids to express HA-tagged muNS fusions.

Construct^a^	Primers^b^ (5′-3′)	Expressed protein^c^
pCINeo-M3(1–154)-HA	F-GCGGAATTCATCATGGCGTCAACCAAGTGG	muNS(1–154)-HA
pCINeo-M3(1–154)-HA	R-GCGTCTAGATTACGCATAATCCGGCACATCATACGGATAATCGGGGGAATCAGCGGTGG	(region 1b)
pCINeo-M3(1–380)-HA	F-GCGGAATTCATCATGGCGTCAACCAAGTGG	muNS(1–380)-HA
pCINeo-M3(1–380)-HA	R-GCGTCTAGATTACGCATAATCCGGCACATCATACGGATATGGAGACCGTCTAGCGAGAAG	(region 1c)
pCINeo-M3(1–448)-HA	F-GCG GAATTCATCATGGCGTCAACCAAGTGG	muNS(1–448)-HA
pCINeo-M3(1–448)-HA	R-GCGTCTAGATTACGCATAATCCGGCACATCATACGGATATGGACCAACGGACGAATCG	(region 1)
pCINeo-M3(1–477)-HA	F-GCGGAATTCATCATGGCGTCAACCAAG TGG	muNS(1–477)-HA
pCINeo-M3(1–477)-HA	R-GCGTCTAGATTACGCATAATCCGGCACATCATACGGATATTCCCGAGCAGGTTGAACATC	(region 1+2)
pCDNA3.1/Zeo-M3(539–635)-HA	F-GCGGAATTCATCATGGCGCGTGTGGAAGCGTTAAACCAAG	muNS(539–635)-HA
pCDNA3.1/Zeo-M3(539–635)-HA	R-GCGTCTAGATTACGCATAATCCGGCACATCATACGGATACAGATCATCCACCAATTCTTC	(region 4+5)
pCDNA3.1/Zeo-M3(539–605)-HA	F-GCGGAATTCATCATGGCGCGTGTGGAAGCGTTAAACCAAG	muNS(539–605)-HA
pCDNA3.1/Zeo-M3(539–605)-HA	R-GCGTCTAGATTACGCATAATCCGGCACATCATACGGATAGACACGTGTCGCACGACTCATC	(region 4)
pCDNA3.1/Zeo-M3(477–542)-HA	F-GCGGAATTCATCATGGAAGATCACTTGTTGGCTTATC	muNS(477–542)-HA
pCDNA3.1/Zeo-M3(477–542)-HA	R-GCGTCTAGATTACGCATAATCCGGCACATCATACGGATACGCTTCCACACGGGGTTCCCAC	(region 3)
pCDNA3.1/Zeo-M3(381–448)-HA	F-GCGGAATTCATCATGCCATCCTTCTTACTCGGTG	muNS(381–448)-HA
pCDNA3.1/Zeo-M3(381–448)-HA	R-GCGTCTAGATTACGCATAATCCGGCACATCATACGGATATGGACCAACGGACGAATCG	(region 1a)

a Each construct was designed to contain the portion of the M3 gene encoding the indicated amino acid residues of muNS.

b For each truncation construct, reverse primer (R) is in the reverse orientation relative to the coding strand, and the added stop codon and the added HA sequence are double underlined, and forward primer (F) is in the forward orientation relative to the coding strand, and the added start codon is double underlined. The EcoRI and XbaI restriction sites added near the 5′ end of each primer are single underlined.

c Each construct was designed to express a truncated muNS protein comprising the indicated amino acid residues. In brackets, muNS regions as termed in results.

### Construction of recombinant baculoviruses

All the recombinant baculoviruses were generated using the Bac-to-Bac system (Invitrogen, Barcelona, Spain) following the supplier protocols. The construction of the recombinant baculoviruses Bac-muNS, which expresses full-length muNS, and Bac-muNS-Mi, which expresses muNS residues 448 to 635, have been described previously [11].

#### i) Bac-GFP-IC

To express GFP-IC in insect cells, the GFP-Xacs-muNS(477–542)-coding sequence of the pEGFP-C1-Xacs-muNS(477–542) plasmid was amplified by PCR using the forward primer 5′-GCGGGATCCACCATGGTGAGCAAGGGCGAG-3′ (BamHI site is single underlined and the start codon is double underlined) and the reverse primer 5′-GCGTCTAGATTACGCTTCCACACGGGGTTCCCAC-3′ (XbaI site is single underlined and the stop codon is double underlined). The PCR product was digested and cloned into the BamHI and XbaI sites of pFastBac1.

#### ii) Bac-GFP

To generate a recombinant baculovirus expressing the GFP protein, the GFP coding sequence of the pEGFP-C1 plasmid was amplified by PCR using the forward primer 5′-GCGGAATTCACCATGGTGAGCAAGGGCGAG-3′ (EcoRI site is single underlined and the start codon is double underlined), and the reverse primer 5′- GCGTCTAGATTACTTGTACAGCTCGTCCATGCC-3′ (XbaI site is single underlined and the stop codon is double underlined). The PCR product was digested and cloned into the EcoRI and XbaI sites of pFastBac1.

#### iii) Bac-Luc

To generate the recombinant baculovirus expressing the firefly Luciferase (*Photinus pyralis*), the Luciferase coding sequence was amplified by RT-PCR using the Luciferase RNA as template (Promega, Madrid, Spain), and the following primers: the forward primer was 5′-GCGGGATCCATCATGGAAGACGCCAAAAAC-3′ (BamHI site is single underlined and the start codon is double underlined), and the reverse primer was 5′-GCATAAGAATGCGGCCGCTTACAATTTGGACTTTCCGCCC-3′ (NotI site is single underlined and the stop codon is double underlined). The PCR product was digested and cloned into the BamHI and NotI sites of pFastBac1.

#### iv) Bac-Luc-IC

To express Luc-IC, the recombinant plasmid pGEMT-M3 [9] was subjected to PCR amplification with the following primers. The forward primer was 5′-GCATAAGAATCTCGAGATCATGGCGGAAGATCACTTGTTGGCTTATC-3′ (XhoI site is single underlined) and the reverse primer was 5′-GCATAAGAATAAGCTTTTACGCTTCCACACGGGGTTCCCAC-3′ (HindIII site is single underlined and the stop codon is double underlined). The resulting amplified product was digested with XhoI and HindIII and then ligated to the pFastBac1 vector that had been cut with the same enzymes, to generate the recombinant plasmid pFastBac1-muNS(477–542). The recombinant plasmid pFastBac1-Luc-IC was generated by PCR-amplification using pFastBac1-Luc as template, and the following primers: the forward primer was 5′-GCGGGATCCATCATGGAAGACGCCAAAAAC-3′ (BamHI site is single underlined and the start codon is double underlined), and the reverse primer was 5′-GCATAAGAATGCGGCCGCCAATTTGGACTTTCCGCCC-3′ (NotI site is single underlined). The PCR product was digested and cloned into the BamHI and NotI sites of pFastBac1-IC to generate pFastBac1-Luc-IC.

#### v) Bac-GFP-muNS and Bac-GFP-muNS-Mi

To generate recombinant baculoviruses expressing GFP-muNS and GFP-muNS-Mi, the GFP-muNS(448–635) coding sequence of the pEGFP-C1-M3(448–635) plasmid [11] or the GFP-muNS coding sequence of pEGFP-C1-M3 plasmid [9] were amplified by PCR using the forward primer 5′-GCGGGATCCACCATGGTGAGCAAGGGCGAG-3′ (BamHI site is single underlined and the start codon is double underlined) and the reverse primer 5′-GCGTCTAGATCACAGATCATCCACCAATTCTTC-3′ (XbaI site is single underlined and the stop codon is double underlined). The PCR products were digested and cloned into the BamHI and XbaI sites of pFastBac1.

The pFastBac1 recombinant constructs were then used to generate the corresponding recombinant baculovirus, according to the supplier's protocol. The correctness of the constructs was confirmed by DNA sequencing.

### Baculovirus expression and inclusion purification

Sf9 insect cells growing in suspension were infected with 5 PFU/cell of the different recombinant baculoviruses, as indicated in the figure legends, and incubated at 27°C for 72 h. Cells were then pelleted by centrifugation for 10 min at 1000×g, resuspended in hypotonic buffer (10 mM Hepes pH: 7.9, 10 mM KCl, 1.5 mM MgCl_2_) and placed on ice for 15 min. The extracts were then centrifuged at 4°C for 10 min at 2,000×g, and the pellets were washed twice with 10 ml of hypotonic buffer. The resulting pellets were resuspended in 10 ml of hypotonic buffer and sonicated (6 pulses at 45 cycles) to break the nuclei and to shear genomic DNA. The sonicated extracts were centrifuged at 4°C for 5 min at 250×g, and the inclusion-containing pellet was washed and centrifuged five times using the same conditions. The final pellet was resuspended in 1 ml of hypotonic buffer. Samples of each purification step were analized by SDS-PAGE and Western blot. For GFP-IC purification, the final pellet was resuspended in 1 ml of hypotonic buffer containing 500 mM NaCl, centrifuged 10 min at 4°C and 16,000×g, and the supernatant was loaded onto a HiTrap™ desalting column pre-equilibrated with hypotonic buffer (GE Healthcare, Madrid, Spain). The eluted material was centrifuged 5 min at 4°C and 16,000×g and the supernatant was incubated with Xa factor (New England Biolabs, Ipswich, England) for 48 h at 4°C. The extract was then centrifuged 5 min at 4°C and 16,000×g and the supernatant loaded on a HiTrap Q-Sepharose column pre-equilibrated with hypotonic buffer in order to purify the GFP protein (GE Healthcare, Madrid, España). The column was eluted with increasing concentrations of NaCl. The GFP-containing fractions eluted from the column after a 400 mM NaCl wash. Luc-IC-containing inclusions were similarly purified, with the differences indicated on the text.

### Determination of Luciferase activity

Sf9 insect cells were infected with 5 PFU/cell of the different recombinant baculoviruses and incubated at 27°C for 72 h. Then, the cells were lysed, and Luciferase activity of the cells extracts was determined with a Luminoskan-ascent luminometer (Thermo, Waltham, Massachusetts, USA). Results of six replicates are expressed as the mean relative light units (R.L.U.) per well ± the standard deviation. During the purification of Luc-IC, samples of each purification step were diluted 1/200 before Luciferase activity was determined.
